# Transscleral Diode Photocoagulation of Large Retinal and Choroidal Vascular Lesions

**DOI:** 10.1371/journal.pone.0039340

**Published:** 2012-07-09

**Authors:** Yun Feng, Zhizhong Ma

**Affiliations:** Ophthalmology Department, Peking University 3rd Hospital, Peking University Eye Center, and Key Laboratory of Vision Loss and Restoration, Ministry of Education, Beijing, China; University of Tennessee, United States of America

## Abstract

**Background:**

Transscleral retinal photocoagulation with a diode laser is used in glaucoma refractory to medical and surgical treatment. Our main research question was how the technique performed in large vascular lesions associated with hemangiomas of the retina and choroid.

**Methodology/Clinical Findings:**

Patient charts were retrieved from the hospital files for patients who underwent the procedure and were followed for at least 24 months. Five patients (6 eyes) fit the criteria. Cases included Von Hippel’s disease (2 eyes), Coats’ disease (1 eye) and choroidal hemangioma (3 cases). Transscleral diode laser treatment was performed under retrobulbar and topical anesthesia with a retinopexy probe (IRIS DioPexy, IRIS Medical Instruments, Mountain View, CA) applied transsclerally under indirect ophthalmoscope visualization. We found an improvement in best-corrected visual acuity at 24 months postoperatively.

**Conclusions/Significance:**

Transscleral photocoagulation may have a clinical application in these diseases as an alternate to the high cost of photodynamic therapy with photosensitizing agents.

## Introduction

Vascular tumors of the retina and choroid are a major source of long-term visual disability. [1] Contact transscleral photocoagulation with a diode laser is a well-established and relatively safe and effective intervention for glaucoma refractory to management by trabeculectomy or cyclocryocoagulation. Transscleral photocoagulation has been reported in the treatment of diabetic retinopathy, [2], [3] retinal breaks, small retinoblastomas, [4] retinopathy of prematurity (ROP), [5], [6] and proliferative sickle cell retinopathy [7].

The overall goal was to determine if the novel use of this technique warrants further consideration as a routine therapy for these diseases. We found it to have a potential option as an alternative to photodynamic therapy (PDT) when PDT is unavailable due to its high cost for some patients.

## Methods

We conducted a study of the treatment and outcomes of patients with hemangiomatous disease of the retina or choroid and treated with photocoagulation delivered transsclerally under indirect ophthalmoscopic visualization in our facility by the same surgeon (ZM), and followed for at least 24 months. Basic information of the patients is shown in Table 1 including previously medical management, severity of the retinal detachment, treatment parameters, and laser energy with durations and complications.

The records of patients with retinal or choroidal hemangiomas who were treated with transscleral photocoagulation were retrieved from the hospital files. Patient and surgical data were gathered and summarized.

Laser photocoagulation was administered transscleraly with the DioPexy retinopexy probe (IRIS DioPexy, IRIS Medical Instruments, Mountain View, CA, USA) under retrobulbar and topical anesthesia and observed with indirect ophthalmoscopy. An incision was made into the conjunctiva in the quadrant where the lesion was located and the retinopexy probe was inserted through the incision until it contacted the tissue. Laser power was initially set at 300 milliwatts (mW) for 30 milliseconds (ms) and then gradually increased to 1000 to 2000 mW at 2000 ms as needed to achieve tissue ablation. The endpoint of treatment was whitening of the mass. The laser was used to make evenly spaced spots. No patients required a scleral flap.

Obliteration of the mass was confirmed by clinical observation, fluorescein angiography and optical coherence tomography (OCT) at each follow-up. Best-corrected visual acuity was obtained preoperatively and at each follow-up visit (Table 2). Patients were followed at week 1 and months 1, 3, 6, 12 and 24.

**Table 1:** Basic informations of these patients and clinical outcome.

case	diagnosis	age	sex	Previous medical management(times)	Severity of Retinal detachment	Medical management	Treatment parametersLaser energy (mW), duration(seconds)	complications
1	**Von hippel’s disease(OD)**	14	F	PDT (2), PPV(1), LP(1)Avastin intra vitreous injection(1)	Pan retinal detachment	Trascleral diode laser	1400 mW–2000 mW2000 ms	Little Vitreous hemorrhage(totally absorbed 1 week postoperatively)
	**Von hippel’s disease(OS)**	14	F	LP(1)	No retinal detachment	Trascleral diode laser	1000 mW1000 ms	none
2	**Coats’ disease**	5	M	none	Total RD with massive exuadates	Trascleral diode laser	1000 mW–2000 mW2000 ms	none
3	**Choroidal hemangioma**	29	M	PDT(1)	A secondary RD, foveal involved	Trascleral diode laser	2000 mW2000 ms	none
4	**Choroidal hemangioma**	52	F	PDT(1)	Serous RD, foveal involved	Trascleral diode laser	1000 mW–2000 mW2000 ms	none
5	**Choroidal hemangioma**	36	M	none	A secondary RD, foveal not much involved	Trascleral diode laser	1500 mW2000 ms	none

PPV = pars plana vitrectomy, LP = intraocular argon laser photocoagulation, PDT = photo dynamic treatment, RD = retinal detachment.

The study was approved by Peking University Third Hospital’s Institutional Review Board. Written informed consent was obtained from all participants or their legal guardians before surgery. All data for the current study were analyzed anonymously.

## Results

A total of 5 patients (6 eyes) presented with Von Hippel’s disease, Coats’ disease or choroidal hemangioma. Patients ranged in age from 5 to 53 years.

Case 1 is a 14-year-old female diagnosed with bilateral Von Hippel’s disease and a retinal detachment in her right eye. The patient reported gradually decreased visual acuity in her right eye over an 8-month period. Prior to transscleral photocoagulation, she had already undergone bilateral PDT, unilateral argon laser photocoagulation, unilateral intravitreal injection of Avastin (Genentech Inc, California, USA), bilateral cryotherapy procedures and unilateral pars plana vitrectomy for severe vitreous hemorrhage after cryotherapy.

After the written informed consent was obtained from the patient and her parents, transscleral treatment with the diode laser was performed. Laser energy power was delivered at 1400 to 2000 mW for 2000 ms (right eye) and 1000 mW for 1000 ms (left eye).

**Table 2:** Best Corrected Visual Acuity (BCVA) at different time of following-up.

Case	Presenting VA(screening)	First treatment(< = 1 week)	1 mos±1 w	3 mos±1 w	6 mos±1 w	12 mos±1 w	24 mos±1 w
1. Von hippel’s disease(OD)	FC/10 cm	FC/10 cm	20/2000	20/100	20/50	20/50	20/50
Von hippel’s disease(OS)	20/20	20/20	20/20	20/20	20/20	20/20	20/20
2. Coats’ disease	20/2000	FC/10 cm	FC/30 cm	20/100	20/70	20/50	20/50
3. choroidal hemangioma	20/40	20/40	20/30	20/25	20/20	20/20	20/20
4. choroidal hemangioma	FC/10 cm	FC/50 cm	20/2000	20/100	20/100	20/100	20/100
5. choroidal hemangioma	20/25	20/30	20/25	20/20	20/20	20/20	20/20

**Figure 1:**
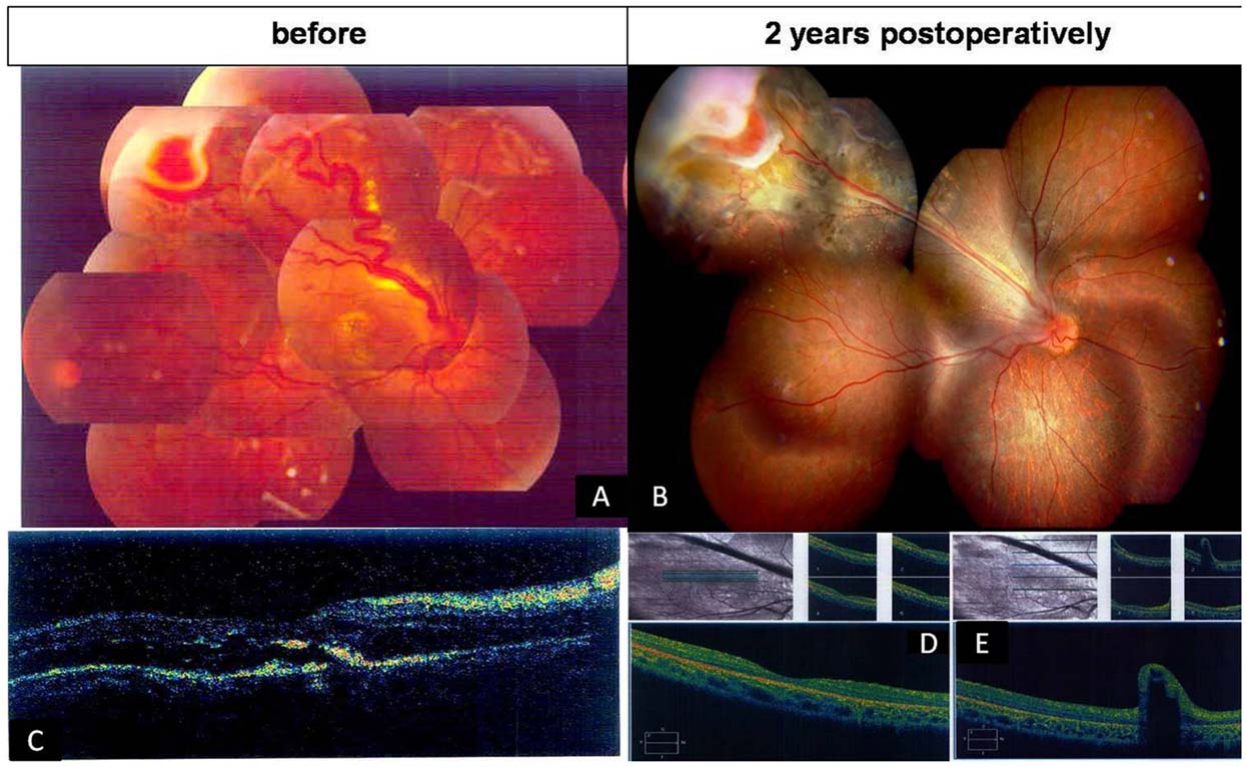
Fundus photographs and OCT before surgery and 2 years postoperatively in a 14-year-old girl with von Hippel’s disease in her right eye (Case 1) (A–E). A. Fundus photograph shows that the retinal capillary hemangioma appears as a red to orange tumor arising within the retina with large-caliber, tortuous afferent and efferent retinal blood vessels. The lesion is surrounded by yellow-white retinal and subretinal exuadates that seem to have a predilection for foveal involvement. C. Cystoid macular degeneration was found in OCT, and the subretinal exuadates was noted. B.The retina is well attached two years postoperatively. Scar formation was noted at the original lesion, previously tortuous vessels were stretched. D and E. the OCT reveals that the macular area is relatively normal and the retina is flat at the region of the stretched vessel at 2 years postoperatively.

**Figure 2:**
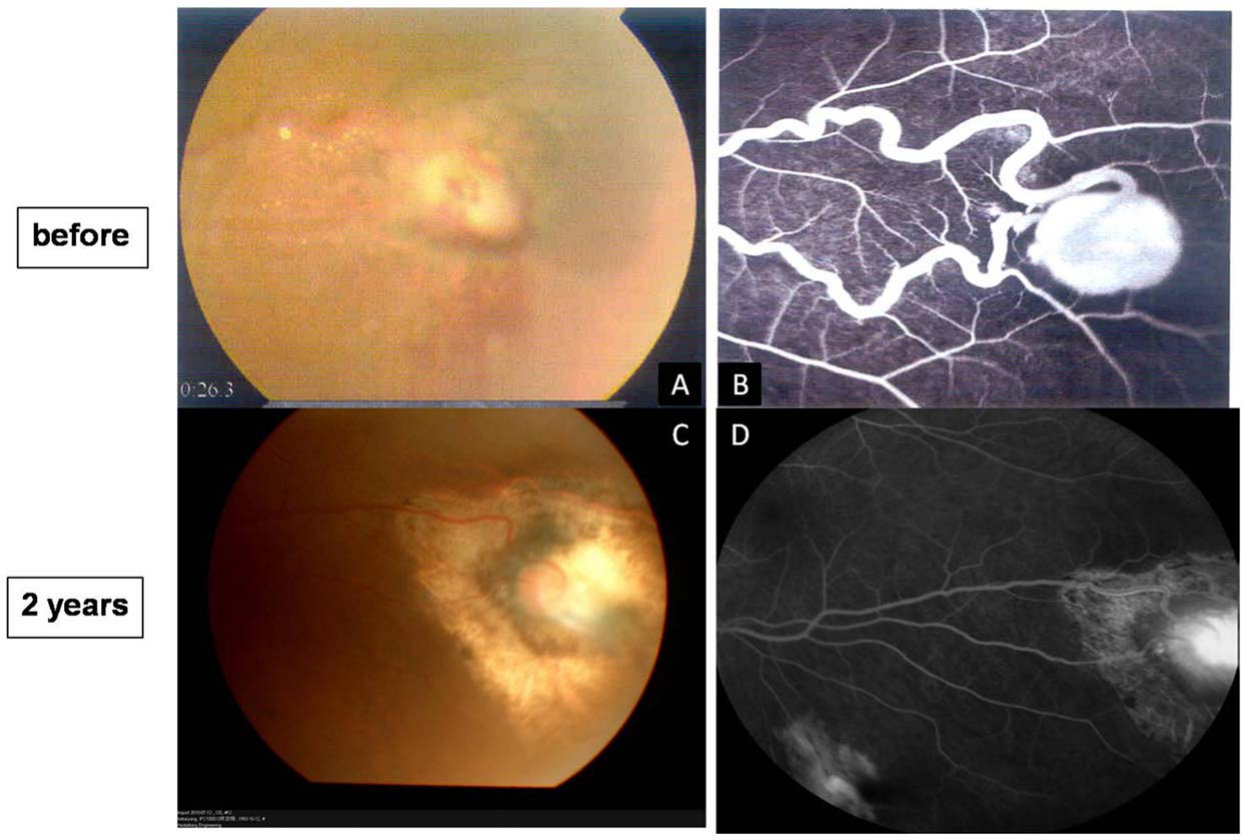
Fundus photographs and fluorescein angiography before surgery and 2 years postoperatively in the 14-year-old girl of von Hippel’s disease in her left eye (case 1) (A–D). A. Left eye of the same patient with von Hippel’s disease (right eye shown in Figure 1). One localized abnormal bean-like lesion is noted at the temporary inferior quadrant with the toutuous afferent and efferent vessels, some yellow exudates are visible surrounding the lesion. B. Fluorescein angiography reveals the hyperfluorescein of the lesion with toutuous vessels at the same area. C. Scar and surrounding chorioretinal atrophy can be seen at 2 years postoperatively, the vessels are stretching to straight and the surrounded exudates are completely obsorbed. D. Fluorescein angriography in the same area at 2 years postoperatively. The chorioretinal atrophy is observed.

Fundus examination showed multiple retinal capillary hemangiomas, supplied by large, tortuous afferent and efferent blood vessels, occurred in the peripheral retina in both eyes. Figure 1 shows serum leakage from the hemangiomas and vessels with their extensive retinal exudates with foveal involvement on the right eye at the preoperative fundus examination and their appearance at 2 years postoperatively in the right eye. Figure 2 shows the fundus examination and fluorescein angiography of the localized lesion in the peripheral retina preoperatively and the resolution at 2 years on the left eye. A small vitreous hemorrhage occurred during the procedure (Table 1). The blood was totally absorbed at 3 weeks postoperatively. Before surgery, the patient’s best-corrected visual acuity (BCVA) was finger counting at 10 cm in the right eye and 20/20 Snellen in the left eye. The right eye improved to 20/100 at 3 months and 20/50 at the 6, 12 and 24-month visits. The left eye was 20/20 preoperatively and did not lose any BCVA at any visit (Table 2).

**Figure 3:**
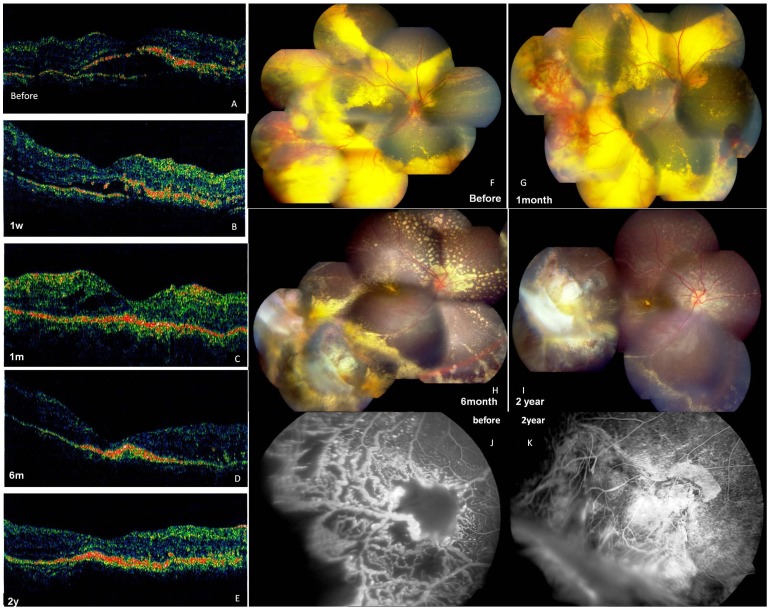
Coats’ disease in a 6-year-old boy: OCT (A–E), fundus photographs (F-I) and fluorescein angiograph at different follow-up durations (case 2). A. OCT reveals the detachment of the neuroretina layer in fovea before the treatment of the surgery. B. the subretinal fluid was partly absorbed and some exudates are observed at one week postoperatively, C.cystoid macular edema was seen at one month postoperatively. D. the subretinal fluid was completely absorbed at 6 months postoperatively. D. the structure is relatively normal in the fovea at two years postoperatively. F. Fundus photographs shows retinal telangiectasias, “light bulb aneurysms”, beading of vessel walls, capillary dilatation, and massive retinal edema with shallow exudative retinal detachment, surrounded by massive white-yellow masses with blurred outlines. G.Fundus photograpy at 1 month postoperatively, there was no much improvement observed after the surgery, except for the dilation of the abnormal vessels. H. The detached retina was reattached with the absorption of the subretinal exudates at six months postoperatively. I. The yellow exudates almost completely absorbed at two years postoperatively. G. Fluorescein angiography at the lesion area. The remarkable retinal vascular telangiectasis, dilated, partly looped, partly beaded thickened vessels and nonperfusion were observed before the surgery. H. Fluorescein angiography at the the same area, significant chorioretinal atrophy was seen without any typical lesion left at two years postoperatively.

Case 2 is a 4-year-old male with Coats’ disease (Figure 3). His parents reported that the boy complained of vision loss and he repeatedly covered one eye with his hand. The patient presented with total retinal detachment with extensive exudates. Upon examination, retinal telangiectasia was observed in the temporal superior periphery and a large retinal detachment surrounded by subretinal yellow exudates was observed in the right eye. Fluorescein angiography revealed retinal vascular telangiectasis, areas of capillary distention and nonperfusion, vessel bleeding and light bulb aneurysms characteristic of Coats’ disease (Figure 3, J). No anterior segment abnormalities were noted. This was the first surgical management of this child’s disease.

**Figure 4:**
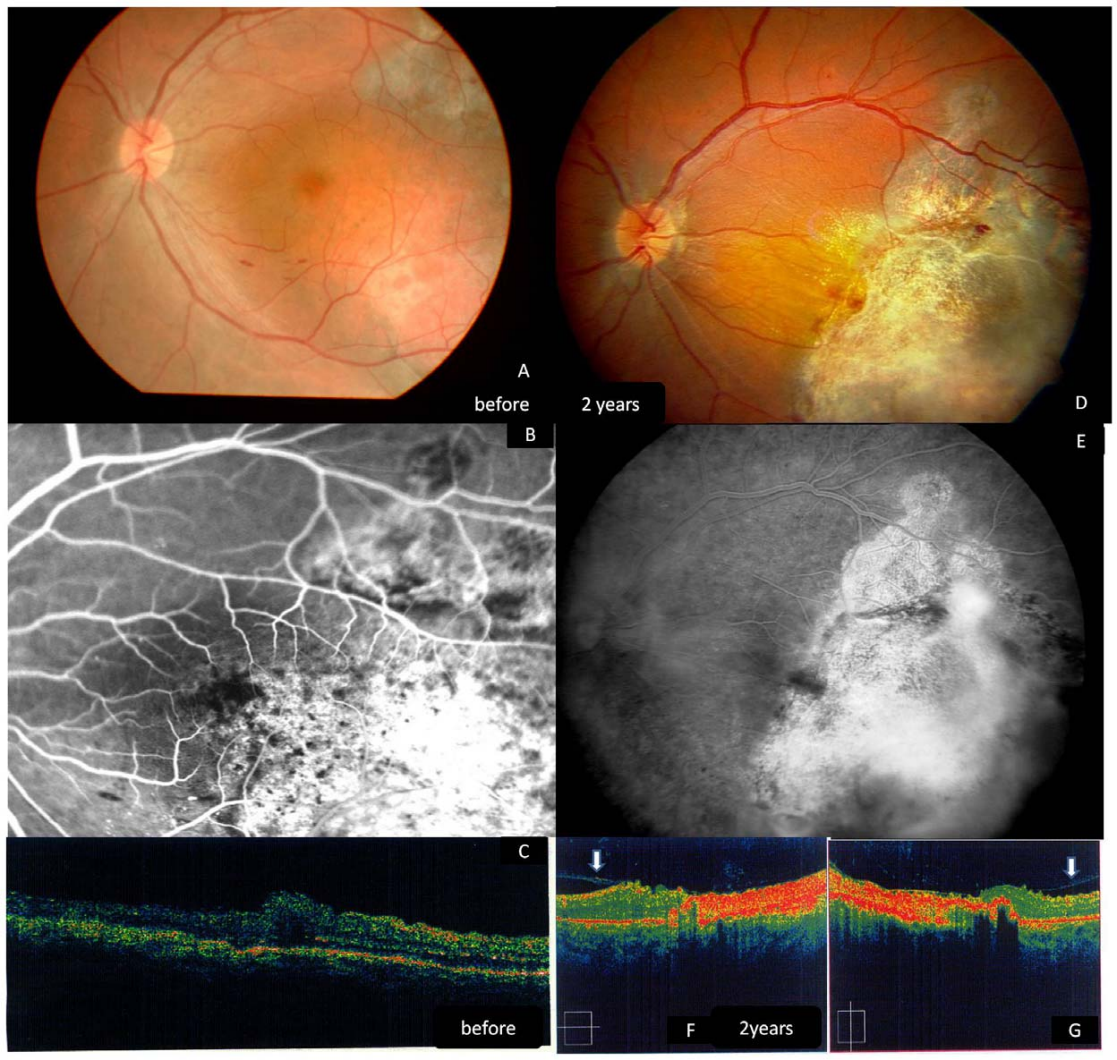
Fundus photography (A, D), Fluorescein angiography (B, E) and OCT (C,F,G) of a 29-year-old man with choroidal hemangioma (Case 3) before our treatment (A–C) and two years postoperatively (D–G). A. massive round orange-colored lesion, slightly elevated at the posterior pole before our treatment. B. Fluorescein angiography reveals that the lesion rapidly develop diffuse hyperfluorescence and do not tend to show the same retinal pigment epithelium changes as melanomas. C. OCT showed the shallow serous retinal detachment. D. Large chorioretinal atrophy and scar were noted two yearspostoperatively. The vessels in the macular area are a bit stretched. E&F, horizontal and vertical scan of OCT revealed an epiretinal membrane (arrow).

Diode laser photocoagulation was achieved with laser energy power delivered at 1000 to 2000 mW for 2000 ms. The procedure was performed without complications. At follow-up, the large subretinal exudates were almost completely absorbed and the patient had good visual acuity (Figure 3, Figure 4 and table 2). Before surgery, the patient’s BCVA was 20/200. At 1 week, it declined to finger counting at 10 cm and at 6 months it improved to 20/70. At 24 months, BCVA improved to 20/50.

Three patients presented with choroidal hemangiomas, vision loss, and hyperopia. Case 3 was a 29-year-old male with a secondary retinal detachment encroaching on the fovea and BCVA of 20/40. Case 4 was a 52-year-old female with serious retinal detachment involving the fovea. Her BCVA at baseline was counting fingers at 10 cm. Both patients had undergone PDT therapy before and suffered of the recurrent of the lesion, but they now sought less expensive treatment. Photocoagulation was achieved with laser settings of 2000 mW for 2000 ms (Case 3), and 1000 mW to 2000 mW for 2000 ms (Case 4). At 24 months, the patient with BCVA of 20/40 improved to 20/20 and the patient with vision limited to counting fingers improved to 20/100.

**Figure 5:**
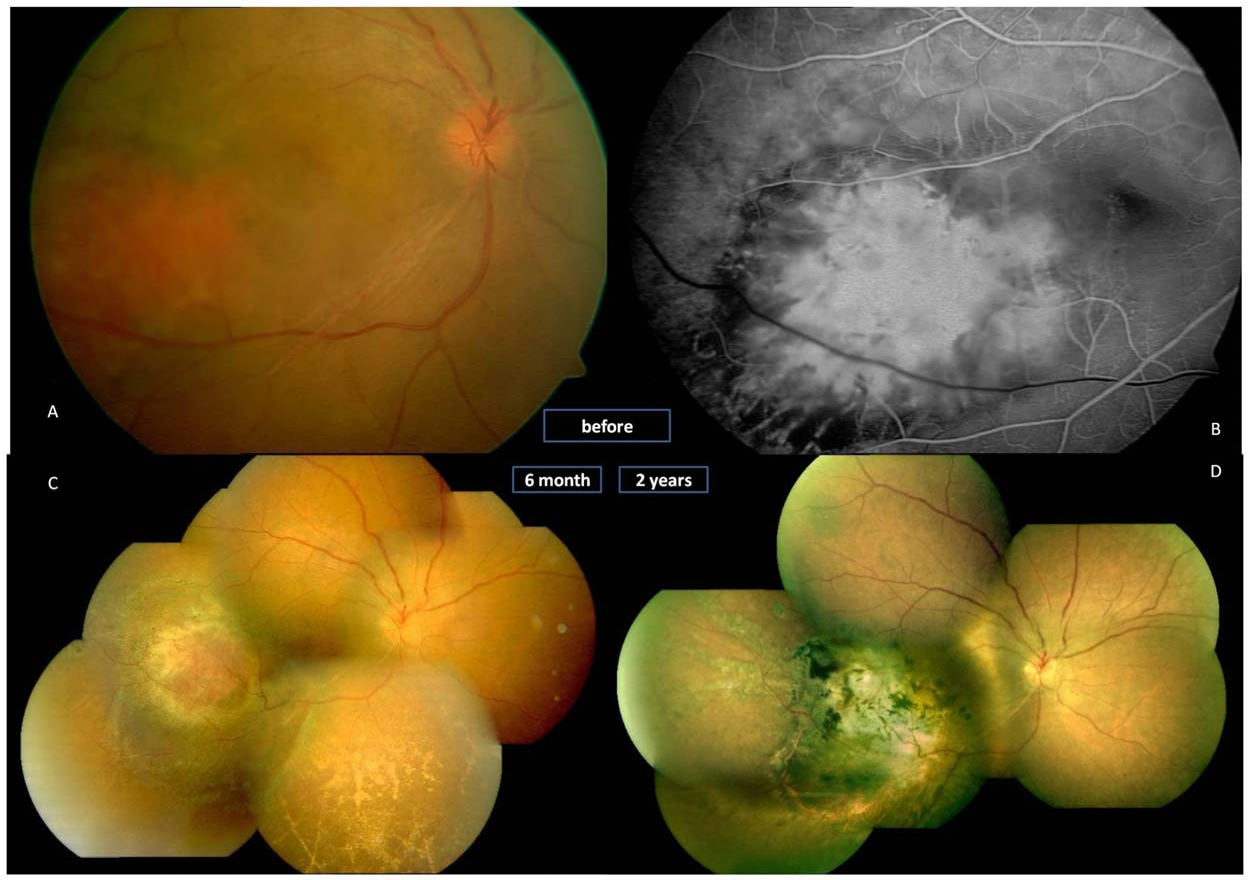
Fundus photography, Fluorescein angiography and OCT of a 52-year-old female with previously failed PDT treatment (Case 4) (choroidal hemangioma) before our treatment and two years postoperatively. A. The lesion appears as a massive red tumor located in the postequatorial zone of the fundus, close to the macular area. B. fluorescein angiography shows the rapid diffuse hyperluorescence at the corresponding area. C. The tumor regressed 6 months postoperatively with a well reattached retina. D.Pigmented chorioretinal scar can be seen at 2 years postoperatively.

**Figure 6:**
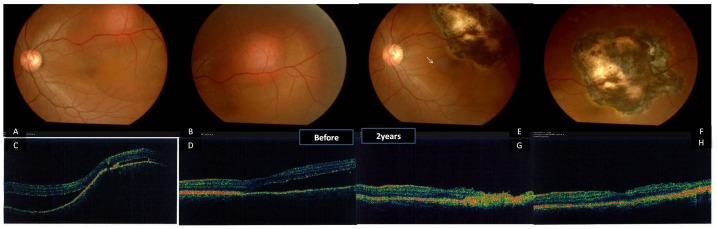
Fundus photograph and corresponding OCT of a 36-year-old male without any previously treatment (Case 5) (choroidal hemangioma) before the surgery and two years postoperatively. A & B. There is a localized red tumor located at the postequatorial zone of the fundus and a secondary retinal detachment that extends into the foveal region.C&D. horizontal and vertical scan of OCT at macular area shows detachment of the outer retinal layers with fovea involved. E&F showed thereattachment of the retina at 2 years postoperatively. The tumor regressed after diode laser treatment, leaving a pigmented chorioretinal scar. The arrow shows a little bit stretching of the retina. G&H. horizontal and vertical scan of OCT at macular area reveals a relatively normal fovea at two years postoperatively.

**Figure 7:**
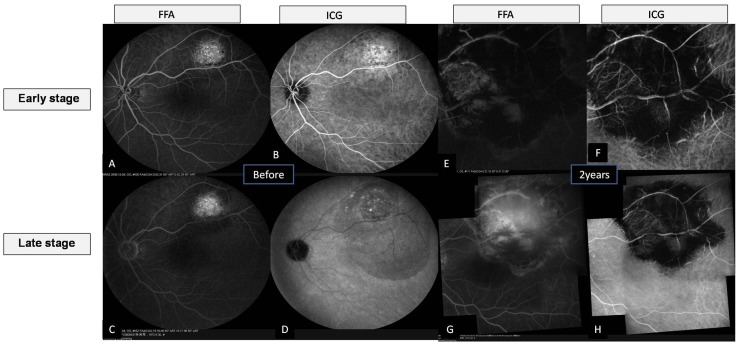
Fluorescein angiography and corresponding ICG findings of Case 5 before the surgery (A–D) and two years postoperatively (E–H). A & B, at early stage, the rapid developed hyperfluorecence of a small localized round lesion reveals a choroidal angioma. C&D. at late stage the secondary serous retinal detachment can be observed. E-G. chorioretinal scar can be noted without any recurrent lesion.

Case 5 was a 36-year-old male with a secondary retinal detachment not much including the fovea and with no previous treatment. He could not afford the PDT treatment. After he was advised about the benefits and risks, he chose the diode laser treatment. Photocoagulation was achieved with a laser setting of 1500 mW for 2000 ms. BCVA was 20/25 preoperatively and 20/20 at 24 months. In all three of these patients, the tumor regressed after diode laser treatment, leaving a pigmented chorioretinal scar. Figures 4, 5, 6, and 7 show the preoperative and postoperative fundus in cases 3 to 5. No evidence of tumor regrowth was observed. Epiretinal membrane was found in one patient (Case 3) by OCT at 6 months postoperatively (Figure 4, arrows). The pigmented scar and retina appeared stable at 2 years postoperatively. No additional treatments were administered to patients with choroidal lesions.

## Discussion

Von Hippel’s disease, Coats’ disease and choroidal hemangiomas are large vascular abnormalities that are difficult to treat. While many of the current approaches such as cryopexy, transpupillary thermotherapy (TTT) are successful for small lesions, treatment of massive lesions is more problematic. Damage to the lesion is problematic and recurrence often follows treatment. PDT therapy is a common treatment, but costly and sometimes repeated treatments are required. Many patients cannot afford PDT therapy, especially in developing countries. The diode laser (810 nm) with the characteristic of high scleral transmission and notable absorption, may be an alternative treatment to those large vascular abnormalities. There were reports on treating small retinoblastoma with transscleral laser [4]. According to this study, larger spot sizes and longer pulse durations could be created by the laser modifications. This led the author to use similar treatment parameters to several cases with massive vascular abnormalities which were successfully managed using transscleral diode laser by D-probe with long-duration, large spot size appears complete since the large spots may result in full-thickness chorioretinal necrosis and closure of the overlying retinal vessels [4].

The transscleral diode laser is a long wavelength laser with a good safety profile and fast learning curve in the management of refractory glaucoma. In our cases, visual acuity improved in 5 eyes and remained at 20/20 in the left eye of the patient with bilateral Von Hippel’s disease. Vitreous hemorrhage occurred in 1 eye and was reabsorbed without further adverse events. Laser power was initiated at a low level of 300 mW and increased to achieve ablation of the blood vessels.

Coats’ disease is a rare idiopathic retinal condition typically found in the first decade of life and results in a gradual loss of vision. Blood leaks from the abnormal vessels and leaving behind cholesterol deposits. Coats’ normally progresses slowly. At advanced stages, retinal detachment is likely to occur. Multiple approaches are available for these diseases including observation, laser photocoagulation, cryotherapy, surgical management, and enucleation. Shields et al [8] analyzed 150 Coat’s disease patients with different treatment options including observation, cryotherapy, laser photocoagulation, various methods of retinal detachment surgery, and enucleation, and developed a classification system. Patients who present with stage 1 to 3 Coats’ disease have the best visual prognosis, and patients with stage 4 or 5 have a poor visual prognosis [8]. Our Coats’ disease patient was stage 3b (total retinal detachment). In this classification, the risk for poor visual acuity is 74% in stage 3 in his study [8]. The changes in our Coats’ disease patient are dramatic: the previous massive subretinal exudates were almost completely absorbed with good visual acuity by only one time treatment. This successful treatment of this serious stage 3b Coat’s disease patient encouraged us to try diode laser prior to vitreous surgery because it is cheaper and easier to perform.

The conventional treatment for Coats’ disease involves cryotherapy to ablate abnormal vessels [9], [10], [11], [12]. The outcome of cryopexy for Coats’ disease has reported effectively in early cases [9], [10], [11], however that is less effective in the case with completely retinal detached [10]. Complications of the cryotherapy such as posterior subcapsular cataract and exudative detachment of the retina progressed after treatment are also reported [10]. Experimental test on animals has suggested that there is a significant breakdown of the blood-retinal barrier with both photocoagulation and cryotherapy, but that it is considerably more severe with cryotherapy [12]. Furthermore, patients sometimes require more than one cryopexy procedure to repair the damage and possibly result in thinning of the scleral. Diode laser performed by the transcleral way theoretically has the advantages on both modalities: surgeons can reach the peripheral lesion and performed as cryotherapy with less damages on the blood-retinal barrier as laser photocoagulation.

Laser photocoagulation for Coats’ disease has been reported in the literature. Schefler et al used an 810 nm infrared laser to treat patients with stage 3 Coats’ disease. [13] In their study, 14 of 17 eyes improved, 2 eyes worsened and 1 eye required enucleation. The difference between their study and ours is the method performed: indirect ophthalmoscopy delivery and 20 diopter lenses. Similar results were reported by Shapiro, using a 532-nm laser for patients with stage 3 Coats’ disease; but 3 patients developed cataracts. [14] Three patients developed cataracts. The investigators attributed the cataract development to the laser energy used or the proximity of the laser application to the lens due to the elevation of the retina [14]. We used the 810-nm infrared laser by transcleral contact mode. The surgeon reported that it was easy to target the lesion and the patients were able to cooperate with the surgeon. Cataract was not observed in our study throughout the 2 years follow up. Further studies could be added to tell if the transcleral way is a safe method of laser application. We expect that patients with large vascular lesions treated with the high energy laser would have lower rates of cataract development.

When we focused on our choroidal cases, the epiretinal membrane was found in one eye by OCT at 6 months postoperatively. The scar and the epiretinal membrane seemed stable, as was the visual acuity. Careful follow-up is necessary. Surgeons should be great care in treating those lesions adjacent to macular area. If the lesions are not in the center, such as the patient in Case 5 (Figure 6 and Figure 7), the transcleral diode laser may be as effective as PDT. We did not do a cost analysis, but we theorize that the transcleral diode laser is a more cost-effective approach than PDT treatment.

### Limitations

This is a small study in only 6 eyes of 5 patients to explore our current experience with the procedure. Our study was limited to patients who underwent the procedure with the same surgeon and were followed for 2 years, when outcomes would be stabilized. More cases and long term data are needed to establish its efficacy and safety. Our experience in this small study suggests that transscleral photocoagulation may play a role in the treatment of large vascular lesions in retinal and choroidal vascular diseases and is worth further study.

The overall goal was to determine if the novel use of this technique warrants further consideration as a routine therapy for these diseases. We found it to have a potential option as an alternative to PDT when PDT is unavailable.
